# Respecting values and perspectives in biobanking and genetic research governance: Outcomes of a qualitative study in Bengaluru, India

**DOI:** 10.12688/wellcomeopenres.17628.2

**Published:** 2023-03-06

**Authors:** Manjulika Vaz, Prasanna Warrier, Calvin Wai-Loon Ho, Susan Bull

**Affiliations:** 1 Health and Humanities, St John's Research Institute, St John's Medical College, Bangalore, Karnataka, 560 034, India; 2 Department of Law and Centre for Medical Ethics and Law, The University of Hong Kong, Hong Kong SAR, China; 3 Ethox Centre and Wellcome Centre for Ethics and Humanities, Nuffield Department of Population Health, University of Oxford, Big Data Institute, Li Ka Shing Centre for Health Information and Discovery, Oxford, OX3 7LF, UK; 4 Department of Psychological Medicine, Faculty of Medical and Health Sciences, The University of Auckland, Auckland, 1142, New Zealand

**Keywords:** Biobank governance, genetic research, qualitative research, ethics, India

## Abstract

**Background:** The promise of biobanking and genetic research (BGR) in the context of translational research towards improving public health and personalised medicine has been recognised in India. Worldwide experience has shown that incorporating stakeholders’ expectations and values into the governance of BGR is essential to address ethical aspects of BGR.  This paper draws on engagement with various stakeholders in the South Indian city of Bengaluru to understand how incorporating people’s values and beliefs can inform policy making decisions and strengthen BGR governance within India.

**Methods:** We adopted a qualitative research approach and conducted six focus group discussions with civil society members and seven in-depth interviews with key informants in BGR, identified through a targeted web search and snowballing methods, until data saturation was reached. Data were thematically analysed to identify emergent patterns.

**Results: **Specific themes relating to the ethics and governance of BGR emerged. Fears and uncertainty about future sample and data use, possibilities of discrimination and exploitation in the use of findings and the lack of comprehensive data protection policies in India along with expectations of enhanced contributor agency, control in future use of samples and data, benefit sharing, enhanced utility of samples, sustained BGR and public good, reflected tensions between different stakeholders’ values and beliefs. Fair governance processes through an independent governance committee for biobanks and a system of ongoing engagement with stakeholders emerged as best practice towards building trust and respecting diversity of views and values.

**Conclusions:** Ensuring public trust in BGR requires listening to stakeholders’ voices, being open to counter narratives, and a commitment to long term engagement embedded in principles of participatory democracy. This is central to a ‘people-centred governance framework’ involving a negotiated middle ground and an equilibrium of governance which promotes social justice by being inclusive, transparent, equitable, and trustworthy.

## Introduction

The growing number of biobanking and genetic research projects (BGR) over the last two decades has sparked debate about ethical challenges at the individual and population levels, accompanying the use of stored human biological material (HBM) samples and associated data ^1^. A major concern relates to appropriate consent models, given that all possible uses of sample and data may not be clear at the time of collection, and the limited understanding that the public has about BGR ^2– 4^. In addition to this, issues of ownership, benefit sharing, sample and data sharing, privacy and confidentiality are difficult to address in a one-time blanket or open-ended consent process ^3, 5, 6^. These factors emphasise the need for governance systems to mitigate ethical concerns related to BGR ^1^.

The promise of BGR, in the context of translational research towards improving public health and personalised medicine, has been recognised in India, by both the government and commercial entities ^7, 8^. The Indian Genome Variation Database (IGVdb), IndiGen programme and Genome India project are clear indications of large-scale efforts oriented towards achieving these goals that are funded by the Indian government ^9– 13^. While drug trials are governed by the Drugs and Clinical Trials rules under the 1940 Act ^1^, there is no specific law on BGR in India. The regulatory landscape for BGR in India is primarily defined by rules and guidelines established by the Indian Council for Medical Research’s (ICMR) National Ethical Guidelines for Biomedical and Health Research Involving Human Participants (NEG), and the Department of Biotechnology’s (DBT) draft Biological Data Storage, Access and Sharing Policy of India ^14, 15^. The regulatory infrastructure is expected to be strengthened when proposed statutes like the Personal Data Protection (PDP) Bill, 2019 ^16^, and DNA Technology (Use and Application) Regulation (DNA Technologies) Bill, 2019 ^17^, become law.

The NEG 2017, compared to the 2006 NEG, has an entire chapter dedicated to Biological Materials, Biobanking and Datasets. This provides detailed descriptions of various types of consent processes (including blanket, broad, specific, dynamic, reconsent) and waivers. Documentation of broad consent may be, combined with a multi-layered, multiple option consent format, especially for secondary use of stored samples/ datasets, with additional clauses on financial or non financial benefit sharing. To safeguard confidentiality and privacy of donors of biological material and data, the practices of anonymization, coding and de-identification have also been discussed. In addition to ethical review and oversight by Research Ethics Committees’ (RECs), the NEG mandates that biobanks have a governance structure with a ‘technical authorisation committee’ to oversee the collection and disbursement of stored biological materials and data to researchers ^14^. In spite of these explanations, much ambiguity remains owing to the complexities of different research designs that could be adopted to promote ethical use of, and sharing of benefits from BGR ^18– 20^. Stakeholder engagement could be a means that researchers and RECs could adopt to shape and implement research through the involvement of a wider group of interested stakeholders ^21, 22^. In this connection, the Council for International Organizations of Medical Sciences (CIOMS) in their 2016 Guidelines highlight the importance of equitable distribution of benefits and burdens through community engagement and ethical collaborative arrangements. ^2^ Where community engagement is applicable, there is an added responsibility on researchers involved in BGR to take into account participating communities’ values and preferences in research design and implementation ^14^. Apart from consent and community engagement, data sharing is also encouraged as a means to maximise utility of stored genetic data ^15^. Additionally, the World Medical Association’s Declaration at Taipei on Research on Health Databases, Big Data and Biobanks highlights that vigilance is required to prevent misuse and abuse where biological materials and genetic data are subject to multiple and indefinite uses. ^3^ Under DBT’s policy, researchers are expected to follow the guidelines established by the ICMR in research involving human participants, which are aligned with principles of data sharing and protections of international conventions. However, the implementation of these principles and related considerations within a specific socio-cultural context of India requires careful study given that stakeholders’ expectations and understanding of consent, privacy and confidentiality, secondary research use and data sharing, among other ethical requirements vary greatly across the Indian subcontinent ^1^. The need for rigorous public engagement is clear, as its absence has resulted in repeated controversies in recent laws and government policies in the Indian context. These include the Citizenship (Amendment) Act, 2019 (CAA) ^23^ which empowered the central government to establish a National Registration Authority and maintain a National Register of Citizens (NRC) to collect demographic and biometric data, by which people without certain documentation could lose their citizenship. Similarly, India’s biometric identification programme— ‘Aadhaar’, meant for the targeted delivery of financial and other subsidies, benefits and services, was allegedly being used to determine citizenship and reports of leakages have raised concerns regarding safeguards extended to personal (and sensitive) data ^24– 27^. These national programmes have a bearing on citizen’s ethnicity, as well as data privacy and protection (in the use of biometrics, for instance), and thus have a bearing on biobanking and its governance.

To enable the exploration of trustworthy governance approaches for BGR in India, which are embedded in a system of public engagement, we initially conducted a literature review on worldwide experiences with BGR ethics and governance ^1^. The review revealed valuable modes of collaborative knowledge generation that are culturally situated and engaged. These include ‘co-production’ ^4^, ‘engagement of knowledges’ ^28^, ‘rules of engagement’ ^5^ and relational practice of ‘stewardship’, as seen in examples in Ghana ^6^, Kenya ^7^, and among Māoris ^8^. Based on these insights, the study reported in this paper was subsequently conducted to understand how incorporating people's values and beliefs can inform policy making decisions and strengthen BGR governance within India. The study was conducted in Bengaluru, a fast growing multi-ethnic metropolis with a population of approximately 12 million, and home to several information technology and biotechnology establishments), to understand how incorporating people’s values and beliefs can inform policy making decisions and strengthen BGR governance within India.

## Methods

We adopted a qualitative research approach, guided by the framework method ^29^, in the design of this study. The methods employed in sample selection, data collection and analysis built upon previous work conducted by MV ^19, 20, 30^.

### Ethics clearance

The study was approved by the Institutional Ethics Committee of St John’s Medical College & Hospital (Study No. 208/2019). Additionally, approval was received from the Health Ministry Screening Committee (Ref No. 2019-7913/F1) as this study involved international collaboration. All participants gave written informed consent for participation, audio recording and indicated whether they would like to be recontacted to receive the study’s findings.

### Participants

Purposive and snowball sampling were used to include participants ranging from civil society groups who could represent as well as potentially influence the view of publics, to key informants who were familiar with the process and requirements of BGR. This was complemented by conducting targeted keyword searches on the Google Search engine to identify organisations from which potential participants could be contacted (See Extended data).

Civil society groups included public policy and public health experts, activists and NGO representatives; media and communication personnel; lawyers and data protection advocates; post-graduate students and PhD scholars; and patient interest organisation members. Key informants included genetic and genomic researchers; a biobank manager with experience in immunology research and as a clinician; a rare disease patient; a genetic counsellor; and a clinician with experience as an institutional ethics committee member ( Table 1). All participants held tertiary level qualification.

**Table 1.** Profile of participants.

FGD No.	Area of expertise	No. of participants (M/F/O) Age (median)	IDI No.	Area of expertise/ involvement with BGR (Gender, Age range in years)
1.	Public policy and public health experts	9 (7/2) 57 years	1.	Breast cancer researcher (F, 40-45)
2.	Activists and NGO representatives	7 (2/4/1) 45 years	2.	Bioinformatics / Cancer genomics researcher (M, 30-35)
3.	Media and communications personnel	8 (0/8) 34.5 years	3.	Biobank manager / immunology researcher / clinician (M, 45-50)
4.	Lawyers and data protection advocates	6 (5/1) 36.5 years	4.	Rare disease patient (F, 30-35)
5.	Post-graduate students and PhD scholars	6 (1/5) 32 years	5.	Population geneticist (F, 30-35)
6.	Patient interest organisation members	8 (4/4) 36.5 years	6.	Genetic counsellor (F, 50-55)
			7.	Clinician / IEC member (F, 65-70)
**TOTAL participants**		**44 (19/24/1) 44.5 years**	**7 (M=2, F=5)**	

### Data collection

We organised six focus group discussions (FGDs) and seven in-depth interviews (IDIs) with civil society groups and key informants respectively. Data collection took place between October 2019 and November 2020. The first four FGDs and six IDIs were conducted in person, while the last two FGDs and IDI were conducted remotely using Microsoft Teams in the context of infection control measures for the COVID-19 pandemic. Data collection continued until data saturation on key issues was achieved ^31^. All data were audio recorded after receiving consent.

*In-depth interviews.* IDIs were between 45 to 60 minutes long and provided insight into the practical aspects of BGR governance. Given that key informants were familiar with the processes involved in research, the topic guide for interviews (piloted with the first two interviewees), drew on participants’ experiences related to BGR, specifically the collection and storage of and research on human biological samples and linked data sets for the purpose of genetic and genomic studies. The topics in the guide were developed based on the study objectives and the review of literature ^1^. A copy of the topic guide can be found in the Extended data. 

*Focus group discussions.* Since civil society groups had varying levels of awareness about BGR we drew from the construct of ‘engaging knowledges’ embedded in wider social relations, while facilitating the FGDs ^1, 28^. FGDs lasted between 60 to 90 minutes and began with an overview of BGR—including a short video on a specific Biobank (This video explained in simple terms what a biobank is and how scientists use biobanks to study a wide range of diseases of the 21 ^st^ century enabling prevention and cures). All participants were provided with optional reading material prior to the meeting which covered the international and national regulations on BGR (available in the Extended data). A topic guide was developed on the basis of current literature to direct the flow of the conversation. The topics in the guide were collaboratively developed by the research team to address the study objectives and were informed by the literature review ^1^. It covered participants’ views, perspectives on BGR, their sample and data storage, consent options, access, sharing and return of incidental findings/ results, concerns, and suggestions about better governance of genetic data and stored samples used in secondary research. It was piloted during the first two focus groups and was appropriately fine-tuned. Suggestions emerging from FGDs were documented on a Google Form and e-mailed to participants for individual feedback. These post-meeting analyses helped in calibrating our topic guide for gaps and new areas to probe and contributed towards thematic analyses of the data. A copy of the topic guide can be found in the Extended data.

### Data management and analysis

MV is a social science researcher whose work has focused on the ethics of biobanking in the Indian context, through a lens of justice and fairness, directed at balancing the expectations of potential participants with the interest of researchers and institutions ^30^. MV and PW (a public policy researcher) were responsible for primary data collection and analysis. MV moderated all focus groups with PW functioning as a note taker; PW conducted all individual interviews. Audio recordings of the FGDs and IDIs were transcribed professionally. FGD transcriptions were analysed using NVivo qualitative data analysis software (Version 12, 2018) (alternatively, this analysis can be done using RQDA, the open source and free qualitative data analysis application). IDIs were analysed using the R package for Qualitative Data Analysis (RQDA) software (version 0.3-1, 2018). Codes were generated through a combination of inductive and deductive approaches, then subjected to thematic analysis, guided by the Framework Method ^29^, to identify patterns emerging from the data. Emergent themes were discussed with SB, (an ethicist and a qualitative researcher), and CH, (a legal expert and ethicist) and refined through an iterative process, involving multiple back and forth discussion and changes in the coding structure and emerging themes.

A deliberative multi-stakeholder meeting was planned to share the findings emerging from the analyses and to engage with their feedback. In the context of the pandemic a face-to-face meeting was not possible, and an online meeting was not feasible. Instead, a report of the findings was circulated to consenting respondents, and an extended list of public policy experts. The feedback received has been incorporated into results presented in this paper.

## Results

Specific themes relating to the ethics and governance of BGR have been identified based on the participants’ understanding of, or in some instances experience with, BGR. The agreements and tensions among various participants reflect differing views and values which have important implications for how such resources should be governed. 

### Participants *’* perspectives of BGR

Study participants thought that BGR would be beneficial to society, but governance needed to be strengthened. FGD participants expressed hope about genetics and stated they were willing to participate in BGR if it could contribute towards reducing the burden of disease, informing health expenditure, and advancing the public good. However, they expressed concerns, many of which were expressed with analogous references to programmes such as the Aadhaar, CAA and NRC—particularly experiences related to the collection and use of biometric data, and the tracing of people’s ethnicity—to position their apprehensions related to this kind of research. Overall, key informants focused on the need to improve people’s awareness about BGR, and institute processes to ensure sustainability over the long-term. Examples of these perceptions are provided in Table 2.

**Table 2.** Participants’ hopes and doubts about BGR.

Sub-themes	Illustrative responses	
	Civil Society Groups	Key Informants
*Information* * sources*	“I am a parent of a kid with MPS [Mucopolysaccharidosis]. I have read about biobanking, I'm aware of this history.” (FGD 06)	“See, most people are trying to read on the internet and may be misinformed. So some of the myths have to be demystified.” (IDI 01)
*Varying hopes*	“I feel the benefits of screening for potential disabilities that are genetic is enormous [...] especially with things like autism spectrum disorder, where we don’t even know what it is, that could be a big plus for genetic research.” (FGD 03)	“this research is very valuable to prevent disease like breast cancer, we may get better diagnostic options, better treatment options and better ways of managing the disease” (IDI, 01) “There is a vast divide between rural and urban areas in sample collection. I think it is much easier in rural areas as compared to urban areas ... because there is half knowledge in urban areas [...] you get a little more scepticism or you get more resistance.” (IDI 05)
*Suspicions, fears* * and mistrust*	“I would be happy [contributing to research] if I knew that it would help with the screening [and prevention] of disability or life threatening disease… But my fear is - and my fear is always very radical - can it be used for eugenics or social manipulation.” (FGD 03) “[D]ata have been used as tools of oppression in various contexts - political, social. With Aadhaar we saw what happened. Now it's going into NRC and NPR. There is a lack of understanding of human values in the way we implement things [...] That is where my basic distrust of this biobanking is coming from.” (FGD 03)	“I think there is a lot of suspicion now about the medical profession as to what is happening and you know whether some kind of misuse of tissue is going on [...] They are just scared of being experimental guinea pigs for whatever reason [...] If they feel that the [researcher] is trustworthy, they will [participate]. But if they get the feeling that there is some hanky panky, then that trust goes.” (IDI 07)

***Concerns on the uncertainty about future use and extent of control in BGR.*** Those participants who were knowledgeable about BGR, attributed the utility of biobanks to the fact that they can store large amounts of HBM samples and associated data (including genetic data) and facilitate research over long periods of time. While civil society members supported the availability of anonymised data for research over the long-term, they drew attention to the uncertainties related to biobanking, in terms of access to and future uses of stored samples and data.

“After twenty years what happens; somebody else is in-charge of the research, or something else is happening. How do I control anything? Who is going to access [my samples and data], and for what? There is this whole thing about eugenics and all… What is it that this genomics project is going to do?” (FGD 02)
“[M]y only concern is they [get] consent for some scientific research and later on [use it for] some study they are doing to link to my ethnicity or behavior.” (FGD 05)

The types of consent sought in BGR vary. The researchers in this study stated that they had experimented with several types of consent, and finally settled on broad consent, as it was considered to maximise the use of samples and data over time. However, when consent was first sought, the primary uses of samples and data, as well as their possible secondary uses in research, would be specified. Both civil society participants and key informants noted that it might not be possible to gauge all possible uses of stored samples and data at the point of collection. Concerns were expressed about the lack of knowledge and control participants have over the use of their data, particularly when broad consent has been given to its use. Questions also arose about whether contributed samples and data would be used following the demise of contributors:

“I would like to know how they will store the collected data like how long they will store the data. And after the study, maybe after some years or something, will they destroy the data? Or are they going to store it for so many years… and after my death, will they use my data in such a situation? Will they inform my family, or anyone related to me?” (FGD 05)

One key informant recounted the dilemmas of an ethics committee having to decide whether access to raw data and participant information could be given to research groups, and specifically those with commercial intent or professional gain, three decades after its initial collection.

“I can tell you in the cancer registry itself, I was involved at a time when there was a crisis. …[It] had data from [1980s], which was really solid data… [and] general consent was taken at the time of registration. In [2010–2020], when cancer research became a lucrative field, there were groups who wanted that data. The registry had to really struggle in order to protect that data because, [in addition to] the tables which had [been published], they wanted the raw data, and in fact, they even wanted to go back to those patients… We didn’t know if they were alive or dead. It was not ethical, and we had to really struggle and convince the authorities that it's not right for us to hand over this data.” (IDI 07)

A specific concern raised by a key informant was that misleading conclusions could be drawn owing to attrition in the sample collection process and deterioration of samples over time if they are not stored in proper conditions. Similarly, the lack of standardisation in the way data are organised could also lead to inconsistencies in research over time. Another concern was that associations between genes and diseases may be overemphasised for personal and professional motives.

“With more and more secondary use of data, if your data is not organised or not accurate, then you could see multiple conclusions being drawn [...] publications will come out with incorrect and misleading conclusions […] At the level of research, one risk is to overstate the links between genes and diseases. People think they need to make a big enough case that then leads to them moving forward in their careers.” (IDI 03)

***Cautions about discrimination and exploitation.*** Participating in BGR was seen to be associated with risks that sample and data contributors could face discrimination or exploitation. Civil society participants (specifically the public health experts, legal experts, activists, and patient interest group members) and key informants stated that it was important to mitigate harm to participants and to ensure research was conducted in an equitable manner.

Discrimination linked to outcomes of genetic research and access to genetic data for purposes other than research (such as to inform decisions about insurance, government benefits, criminal charges, and employment), was a fear raised repeatedly by those civil society participants involved with social and media activism. Noting that genetic information is unique to an individual, like biometric data, those already concerned about Aadhaar and NRC data, questioned the feasibility of anonymising DNA data. In addition to individual harm, the possibility of group harm in the context of research involving participants belonging to a particular caste and geographically identifiable communities was articulated in the FGD with lawyers and data protection advocates:

“[I] would be concerned with harm of two types. One is targeted harm, where someone decides to use data against me. For instance, if my insurance provider [has] access to my data through some network and [that may affect my ability to procure a policy]. The other is class-based harm, where it’s decided that a certain marker indicates certain risks and later on [those markers] prove to be incorrect.” (FGD 04)

Concerns were raised that genetic screening (and testing) could become instruments of exclusion, reflecting the debates about Aadhaar. A key respondent also believed the outcomes of genetic research had the potential to become discriminatory, while noting the benefits of genetic research in terms of aiding personalised medicine.

“I think it could come up when trying to exclude tribal or OBCs [other backward classes] from certain services from the Indian government, especially when there is a heavy recessive disorder burden within a specific ethnic group in India. In one way it could be good because it could help [determine the effectiveness of] medication. On the other hand, it could very easily be used for discrimination.” (IDI 05)

Civil society participants were also worried about their samples and data being commercially exploited when they consented to participate in academic research or undertook genetic tests. They believed that people would not know what they are consenting to and would check boxes that permitted further use of their samples and data without realising that they could be used for commercial purposes. These fears were associated with their belief that commercial entities may not be bound by the ethical approvals that research institutions require. Civil society participants’ concerns were reiterated by some researchers who stated that this already occurs in India and abroad, especially when research is funded by a pharmaceutical company, or other entities that can benefit from the outcomes of genetic research.

“We do a genetic test, and there is one tick mark [asking if] you allow the use of samples for further research purposes? Everybody simply signs yes without knowing anything which is again commercially exploited.” (FGD 01)

Inequities in society were also seen to raise risks of further exploitation while accessing benefits of BGR. Other inequalities in research were related to researcher-participant and researcher-clinician relationships, where both clinicians and participants were treated with a lack of respect.

“When there is a new discovery, it is often those who did not contribute as research subjects who seem to be the first beneficiaries [...] Commonly the study will be done in a low income setting but the first people who have access to an actual intervention that came out of that research will be the people who are more well off.” (IDI 03)
“The surgeon knows where to remove the tissue and how to remove the tissue… Very often, the lab researchers treat surgeons very badly… they say that you're only a supplier of the biopsy [...] So there’s a lack of respect from the lab towards us and I have a personal experience of that environment [...] It doesn't affect the relationship with the patient, but it affects the relationship [between the surgeon and] the researcher. And you will have the surgeon just not wanting to contribute towards research.” (IDI 07)

***Addressing agency and control in future use of samples and data.*** Recognising the imbalance of power between research participants and institutional actors such as researchers and funders, civil society voices emphasised their right to know, to withdraw, and in the long run to be forgotten, as significant expectations in BGR. The right to know related to improving people’s awareness about the use of their samples and data in primary and secondary research and enabling them to make an informed decision about whether they wanted to participate for immediate and longer-term periods.

“[I]n a world right now where data is chasing us all over the place, the right to be forgotten - in a research environment or in an experimentation environment where it's the right for your genetic material to no longer be used.” (FGD 03)

Key Informants dealing with large datasets noted the difficulty in enabling a participant to withdraw themselves from a database once data have been anonymised and aggregated. However, when working with de-identified data, they recounted experiences of the biobank requesting them to remove certain participant IDs, when a participant withdrew their consent.

“Right now, I’m dealing with the UK biobank. I just got an email saying participant number [so and so] has withdrawn from the study [...] So what I do is, in my analysis I just discard all those patient ids.” (IDI 05)

When participating in long-term BGR, civil society respondents expected these rights would enhance participants’ awareness, their sense of confidence and reduce chances of exploitation, uncertainty, and lack of control regarding secondary uses of stored samples and data. They wanted more than information sheets and consent forms provided in simple language that research participants understand. The need for engagement with potential research participants prior to taking their consent as well as ongoing engagement beyond initial consent was expressed, to promote trust, and keep participants informed about the use of their samples and data, as well as outcomes of previous research.

“So, consent not just in words, but consent also in other forms, where I think the people or the individuals or the community needs to know what they are giving. So if you can’t spend time on that then don’t take the sample. That’s it. [This] can be worked out in the process of engagement” (FGD 02)

***Authorising data use and maximising utility.*** When consent to BGR was initially sought, civil society participants suggested that purpose limitation should be considered. An underlying dynamic process was alluded to, (rather than broad consent), where proposed research beyond the initial consent is reviewed and additional consent is perhaps sought. Researchers would have a certain degree of freedom to use samples and data without having to recontact participants, while enabling participants to exercise some control over the use of their samples and data. Such an approach could also promote trust and transparency, key values emphasised in sustaining participant involvement in BGR over time.

Participants also believed that consent needed to go beyond initial engagement and be an ongoing process. Examples discussed by researchers included a breast cancer patient support group where a medico-social worker acted as an intermediary between researchers and participants. The social worker regularly engages with participants and helps them manage their disease. This helped researchers be sure that participants made an informed decision about long term involvement in the study.

***Benefit sharing.*** Civil society participants and clinicians wanted their role in BGR to be acknowledged and wished to be more involved in the research process. They also expected to have fair access to benefits from the research. 

*Direct benefits.* Apart from ensuring that research contributed to public good, civil society participants also envisaged some form of individual benefit from research participation. This included the return of test results and any relevant incidental findings. Clinicians noted that this could contribute towards equity and fairness in the research process as well, given the costs associated with genetic testing within India.

“I said that you should keep sharing the results in the interim also [...] That way, it matches with the right to know and there is also this need to know.” (FGD 02)
“I think in some cases, researchers are also duty bound [to return incidental findings] ... You collect blood samples for some research that you are doing, in the process of analysing these blood samples, you find that mine is HIV +ve, in that situation, you would see an obligation to inform me.” (FGD 04)

The interviewed researchers were divided on returning incidental findings to participants. While some thought it was imperative to communicate findings related to known clinical conditions for which treatments exist or conditions that could put other family members at risk, others were hesitant to return incidental findings to participants if it was not explicitly mentioned in the consent form or if they did not have a genetic counsellor on the study.

“It's so obvious when you have this genomic data, you might find some accidental secondary thing. Whether you take this back to the patient or not [...] it's already been informed in the consent form, whether [they] want to hear anything other than what’s been [consented for]. Unless I have a genetic counsellor, I wouldn’t take any information back. We need that person in place in order to take the genetic information back to the patient.” (IDI 02)

*Sharing of commercial benefits.* Other returns and benefits that civil society participants discussed included free access to any intervention that was an outcome of research and monetary benefits from research which led to commercialisation.

“Let’s say it is a pharma company that is using your biobank details and produces some expensive medication for a certain thing. Then they are going to profit out of it. I would want to know why I’m not getting a cut from it.” (FGD 03)

In contrast, researchers stated that participants should be aware that they may not get any immediate benefits from their participation, but rather it is for the progress of science and for public good.

“My interest is to purely understand the tumour [...] and how that information can be [used] to come up with a better drug. My interests are oriented towards that. But if we keep on restricting [research, saying] you can't do this, you have obtained consent only for this so you have to do only this, you can't accomplish this, you can't patent this, then there is no scope for what is happening in the lab to reach the patient at all. So [we need to] reach a point of equilibrium of governance.” (IDI 01)

***Responsible research—Gatekeepers and their roles.*** Biobanks, researchers, and institutional ethics committees were seen by civil society participants, particularly data protection advocates, as custodians of samples and data with a fiduciary responsibility, and accountable to contributors. They were expected to institute mechanisms which ensured the research did not go against deeply held values or beliefs of the contributors and was undertaken in a manner that mitigated potential harm to participants.

“The principal collector of the data is a fiduciary. That means, their primary responsibility is to whom they collect data [from].” (FGD 04)

In contrast, researchers believed that their responsibilities were primarily to regulators and funding agencies. While they recognised researchers’ responsibilities to conduct research ethically, they viewed institutional ethics committee approval and the procurement of funding as an affirmation of ethical acceptability.

“… most of the data that we are working with are all published data [...] we will check whether the study has been ethically approved and based on that we will go about [our research]. When it says that it has been reviewed by the [ethics] committee at the institute then it is kind of trustable.” (IDI 02)

Institutional ethics committees were expected to be especially vigilant while reviewing primary and secondary research proposals and monitoring the conduct of such research. While some believed that ethics committees would represent concerns of the participants and stated that their presence instilled trust, others believed that such committees largely protected research interests and could not exercise sufficient authority to protect participants.

“Because one of the big things about ethics committees is that they lack teeth. They can’t DO anything besides have a nice conversation.” (FGD 03)
“What I would say is that checks and balances are very important in whatever work we do. So definitely, I see a role for the ethics committee for something like that. [They] moderate and ensure that things are happening in the right way [...] when [ethics] committees are there that builds on trust.” (FGD 06)

Civil society participants emphasised that researchers should refrain from conducting studies related to the genetic basis of intelligence, behaviour, and ethnicity. They also stated the need for a systematic process to enable the researchers to vet information being published by the media about their research findings. This was considered important because of the potential for misinterpretation or intentionally spreading misinformation about genetic research findings. A similar view was expressed by a key informant:

“I think there are a couple of things. One, [researchers] should be very careful about the research questions that they try to ask. I think many sensible geneticists will not go down this path of trying to understand genetics of cognition, precisely because there are negative implications and also, we still don’t really have a grasp as to how we should be conveying the genetic information to the public [...] And the second point is, some checks and balances [need to be there when the] researcher is communicating with the media.” (IDI 05)

### Governing future uses of samples and data

Civil society groups and key informants spoke about the lack of comprehensive data protection policies in India and recommended developing stringent data protection legislation with a charter of rights for participants and responsibilities of researchers. To ensure privacy and provide protection against stigma and discrimination on social, economic, and political fronts, participants expressed the need for robust policies that would delineate procedural requirements for biobanks and other entities that may want to access, store, or use genetic data. Additionally, researchers believed that institutional policies needed to be established to define protocols to follow in the event of conflicts among researchers and within research institutions.

“Actually, we have no [data protection] policies. They have to be made robust. Organisation of that data… protecting it… if there are policies [we can] do it in a systematic way [...] We also don’t have policies for data sabotage. So, if two people in the lab fight, and one ends up leaving, or we end up firing them, how do we ensure that whatever data is already in his/her possession does not get misused.” (IDI 01)

Key informants stressed the importance of documents such as memoranda of understanding (MOUs) and material transfer agreements (MTAs) in promoting trust among collaborating researchers and strengthening research governance at an institutional level. However, the limitations of instruments such as MOUs and their potential for misuse was also highlighted.

“It is just an understanding… actually I don’t have control… it’s a matter of trust and we have an MOU… We have drawn an MOU with our referral labs where we have also very specifically mentioned that if there are any issues related to further research on this, my consent should be there as a referring doctor and the patient’s consent.” (IDI 06)
“I think there are loopholes. Usually, they say if the data cannot be analysed in a particular way in India, then you can send it abroad [...] But it's very loose, what do they mean by it can't be analysed here [...] They just send it abroad because it's easier for them to give it to somebody and get their name on big papers. That’s the only reason and then it stays there.” (IDI 05)

While researchers discussed the benefits of MOUs, in terms of collaborating with other scientists and institutions to aid ongoing research, civil society participants and clinicians sought an acknowledgment of people’s contributions to research and ways of overcoming uncertainties related to future use of samples and data. It was suggested that participants could be provided with a certificate or card that could be handed down through the generations so that participants and their families could contact the research institution to get information about the use of their samples and data.

“I would be happy to know, I am not telling [the researchers] that they need to come back, it’s about respecting each other [...] If you gave me…a, you know a card or something [like an account in the] post office. I show it to the person, maybe my third generation would just like to know about it and go there and see that this is what the status is.” (IDI 04)

***Governing biobanks.*** Civil society participants and key informants agreed on the importance of preventing unauthorised access and exploitation of biobank samples and data. Registering biobanks with the government and implementing a legal framework to govern their operation was considered key to achieving this. However civil society participants were also sceptical about the value of biobanks operating exclusively as state-run entities, stating their lack of trust in the government and the possibility of their samples and data being used without consent by law enforcement agencies or for other civil matters and forms of exploitation.

“...the biggest concern that I have is my lack of trust in the systems in this country… data have been used as tools of oppression in various contexts – political, social.” (FGD, 03)
“…it happened … I had not informed the insurance company about my child’s diagnosis …when I go to them, they claim that it is public information...” (FGD, 01)

In reflecting on how biobanks could be sustained and promote the public good, participants reflected about appropriate approaches to funding. Views differed about whether a biobank should be a non-profit entity, relying on grants and philanthropy and whether a non-profit structure would be a deterrent to quality maintenance, long term sustainability, and the advancement of science and innovation.

***Fair governance processes.*** Participants suggested that an independent governance committee for biobanks could specifically function as a fiduciary, monitoring biobanks, reviewing access requests, and managing grievances. Such oversight could enhance the efficiency of biobank governance, augment ethics committee review, control unauthorised uses of data, improve tracking capabilities and ensure ethical compliance. The composition of such a governance committee would need to be transparent and independent of vested interests in the outcomes of the research. This again would help in building public trust in the BGR system.

“There [should be] a committee that reviews the proposal whoever wants to access the data. [After reviewing] how the data is going to be used and [they] give provision to access the data.” (IDI 02)

Finally, to embed varying values and beliefs of the people in robust BGR governance, blanket regulations would not be sufficient. Engagement with participants was seen as a process through which cultural norms and values could be understood, acknowledged, and respected. It was suggested that these could be affixed to datasets as metadata to inform future research. Additionally, engagement would help to formulate mechanisms for the return of findings, possibly share benefits and even a stake in profits with participants, future generations, and communities they belong to.

“[P]eople deserve to have their data handled with dignity… I feel there is a need for a fundamental value system which governs all of this, but custom made to the local needs of the community and their values and beliefs.” (FGD 02)
“Try to involve an anthropologist, sociologist who works with the community to say ‘look if we want to deal with this community what is one way we should be handling it, are there any rules we should be sensitive towards.’” (IDI 05)

## Discussion

This study was part of a larger effort to engage with the public and understand how ethical considerations relating to the conduct and governance of BGR could be addressed in an inclusive, collaborative, deliberative manner ^1, 28, 32– 36^. Our focus groups and key respondents were a microcosm of the urban metropolis of Bengaluru and reflected the dynamics of aspirations and fears of a population that wished to be heard, that spoke up for the marginalised and wanted their interests protected and public good promoted. They were likely to be more articulate and had greater agency than the marginalised or those accessing healthcare in the public health system; the latter being more likely to be recruited as BGR participants. Even so, the contexts of people’s lives and their lived experiences influenced the concerns expressed by participants and brought forth tensions between different stakeholders’ values and beliefs. For civil society members, views arose, to a large extent, from lived experiences with government policies related to biometrics, citizenship, personal data usage and access by commercial firms, and the public’s relationship with the healthcare system. This is often not viewed from a research guideline lens but emerges as a public reality. Researchers were aware of the need for ethical practices in their work and the need to ensure sustained public trust but viewed restrictions on their access to samples and data as limiting their research and the advancement of science. The common values emerging from all stakeholders centred on the importance of increasing participants’ understanding of BGR and that outcomes of BGR promoted the public good. This form of public participation in BGR focusses on systematic communication to increase public awareness about the ethics, and the science and technology of BGR ^1^.

Key expectations of BGR that emerged from this qualitative inquiry were a desire for enhanced agency and engagement beyond initial consent to involvement; to have safeguards in place and upheld by data custodians and ethics review committees; to ensure research was conducted responsibly with limits on potentially stigmatising profiling; and for the implementation of benefit sharing involving the feedback of findings and fair access to research outputs. So, while the NEG prescribes options for broad, blanket and even tiered consent, there is growing public opinion that favours a dynamic consent model where findings of earlier phases of study are shared by researchers while requesting extended consent. This is not intended as a barrier towards further research but a reiteration of people’s stake and support in what is considered BGR towards public good. This process of informing the governance of BGR through eliciting public opinion towards it, is described by world-wide experience as stakeholder consultation or mid-level engagement ^1^.

The findings reported above demonstrate that listening to the voices of diverse stakeholders who are distanced from BGR governance highlights tensions in the current ethical paradigm and elicits interests, priorities, and ideas about governance mechanisms which have the potential to promote trusted and sustainable BGR. These findings suggest that the complexity and heterogeneity of local values and expectations towards BGR should be addressed in a people-centred governance model which seeks to ensure procedural justice by incorporating and respecting the perspectives of a range of stakeholders through an engagement process comprising varying degrees of involvement ^1^. Such an approach builds on the principles of normative stakeholder theory and a participatory paradigm in bioethics, where the concerns and expectations of the various stakeholders involved are balanced in the process of governance ^37^. While in this study, participants were not vulnerable in terms of their socio-economic or educational status, vulnerability of stakeholders within the larger Indian context tends to be construed in terms of ethnicity, socioeconomic status, and educational levels ^1, 38^.

**Figure 1.**
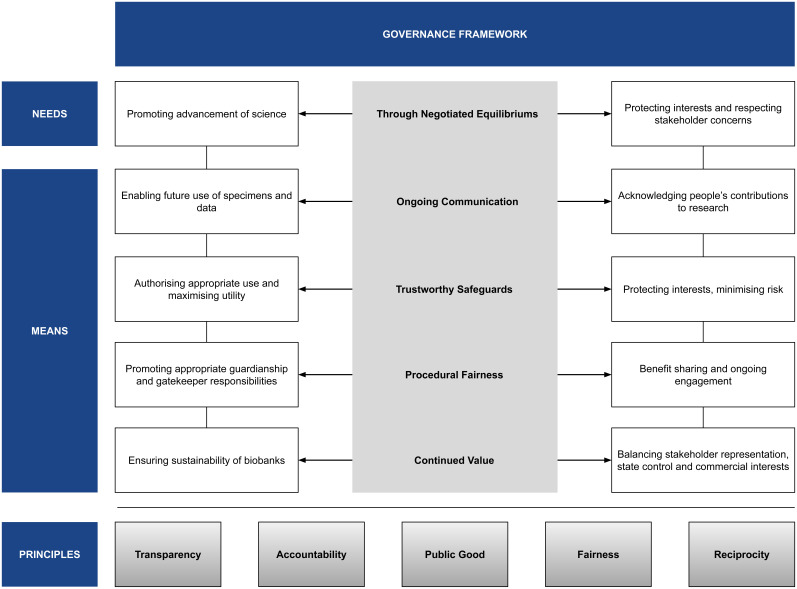
Framework for People-Centred Governance of BGR.

A people-centred approach to governing BGR directed towards a negotiated middle-ground, or ‘equilibrium of governance’, has the possibility to account for varying values and priorities while meeting the needs of advancing science yet respecting local and cultural norms. ( Figure 1). Negotiated equilibriums hinge on ‘counter storytelling’ ^39^, which incorporates narratives of the public and other stakeholders. Counter storytelling has been recognised as an essential element of procedural fairness in a post-colonial environment like India, where vulnerable populations are involved in research, as the present ethical paradigm is researcher-centric, and the findings of genetic research have been used by different groups to drive their own political agenda ^19, 39, 40^.

Suggestions about engaging community voices in governance processes include constituting an independent governance committee for biobanks (as in the ICMR NEG) while also attaching contributors’ cultural norms to datasets seek to uphold the value of respect and echo ideas such as an ‘Ethics and Governance Council’ and ‘Ethical Metadata’ ^3, 41^. Such committees would have a role in supporting scientific research, and in that capacity, could explain why certain decisions were made, even if they did not appear to be consistent with participants’ preferences. The reasons for decisions would be set out in an open manner, and participants could withdraw their consent for that specific research, if they wish. These mechanisms could also help researchers learn of particular sensitivities of contributors and minimise the risks of research that could potentially lead to stigmatisation or discrimination. This dynamic process would be time and effort consuming but has the potential to build long-term trust and trustworthiness of the system.

People-centred governance is particularly important for uncertain future uses of samples and data where there is specific responsibility delegated to ensure that appropriate safeguards are in place. Ongoing communication by such a governance body would reduce such uncertainties as the uses could be communicated on a case-by-case basis. This support system is especially important in the conduct of responsible research—where acknowledging people’s contribution and upholding the dignity of research participants is an intrinsic feature.

### Limitations

The results of this qualitative study were initially intended to inform a consultative engagement exercise where they would be deliberated on by key stakeholders in BGR. The process of facilitated negotiation would have informed recommendations on a governance structure and process for policy adoption. This was not possible in the context of the COVID-19 pandemic, which not only restricted meetings but diverted the attention of government agencies and regulators comprising the key stakeholders in such an engagement exercise.

This study enrolled civil society members and public influencers who were not contributors to a biobank or BGR. They were educated and articulate and not representative of the general population. It was not possible to identify and invite BGR participants to participate in this study as administrators informed us that they had not given consent for their contact details to be shared for such purposes. 

## Conclusion

Our study has reiterated that to ensure public trust in BGR using people’s stored samples and data, listening to stakeholders’ voices and being open to counter narratives is central to a people-centred governance framework based on local values and beliefs. Such an approach seeks to promote social justice by being inclusive, transparent, equitable, and trustworthy. People-centred governance involves negotiating multiple interests and priorities, engaging in regular communication, and ensuring protections and safeguards are implemented by responsible custodians. Addressing imbalances of power, knowledge, and resources; and inbuilt vulnerabilities in communities with complex concerns and expectations cannot be achieved overnight by a governance framework. However, commitment to long term engagement embedded in principles of participatory democracy have demonstrated the value of a people-centred governance approach over time, particularly where vulnerable populations are involved. Best practices for the governance of BGR hinges on addressing potential tensions and competing priorities, while considering common interests in promoting the advancement of science and public good. These lessons offer insight into people centred BGR governance that can be attempted in the context of Bengaluru’s labs and research institutions.

## Data Availability

The data associated with this article is in the form of interview transcripts and group discussion transcripts with multiple personal anecdotes and experiences which cannot be anonymised sufficiently. To ensure confidentiality and privacy of all participants as stipulated in the ethical approval (by the Institutional Ethics Committee of St John’s Medical College & Hospital, Ref No. 208/2019), the data are not made publicly available. Nevertheless, the data will be available to external researchers upon a reasonable request to the first author via an email with a proposal of how the data will be used and a non-disclosure compliance statement. The YouTube video used in this research cannot be shared due to the ethical and copyright restrictions surrounding social media data. The Methods section contains detailed information to allow replication of the study. Any queries about the methodology should be directed to the corresponding author. Harvard Dataverse: Extended Data for Respecting values and perspectives in biobanking and genetic research governance: Outcomes of a qualitative study in Bengaluru, India. https://doi.org/10.7910/DVN/ZUCAU5 ^42^ This project contains the following extended data: Key Word Search -Appendix.docx (keyword searches undertaken to identify study participants) IDI-Topic Guide - Subject Experts.docx (topic guide for in-depth interviews) FGD Topic Guide - Civil Society.docx (topic guide for focus group discussions) Background_Information.pdf (reading materials for focus group discussions) Data are available under the terms of the Creative Commons Zero "No rights reserved" data waiver (CC0 1.0 Public domain dedication).

## References

[1] Warrier P Ho CWL Bull S : Engaging publics in biobanking and genetic research governance - a literature review towards informing practice in India [version 2; peer review: 1 approved]. *Wellcome Open Res.* 2021;6:5. 10.12688/wellcomeopenres.16558.2 PMC11026954 38645686

[2] Beskow LM Dombeck CB Thompson CP : Informed consent for biobanking: consensus-based guidelines for adequate comprehension. *Genet Med.* 2015;17(3):226–33. 10.1038/gim.2014.102 25144889 PMC4336635

[3] Tindana P de Vries J : Broad Consent for Genomic Research and Biobanking: Perspectives from Low- and Middle-Income Countries. *Annu Rev Genom Hum Genet.* 2016;17:375–93. 10.1146/annurev-genom-083115-022456 26905784

[4] Domaradzki J Pawlikowski J : Public Attitudes toward Biobanking of Human Biological Material for Research Purposes: A Literature Review. *Int J Environ Res Public Health.* 2019;16(12):2209. 10.3390/ijerph16122209 31234457 PMC6617000

[5] Hoeyer K : The Ethics of Research Biobanking: A Critical Review of the Literature. *Biotechnol Genet Eng Rev.* 2008;25:429–52. 10.5661/bger-25-429 21412365

[6] McGuire AL Beskow LM : Informed Consent in Genomics and Genetic Research. *Annu Rev Genomics Hum Genet.* 2010;11:361–81. 10.1146/annurev-genom-082509-141711 20477535 PMC3216676

[7] Limaye N : Pharmacogenomics, Theranostics and Personalized Medicine - the complexities of clinical trials: challenges in the developing world. *Appl Transl Genom.* 2013;2:17–21. 10.1016/j.atg.2013.05.002 27942441 PMC5133334

[8] Chakrabarty S Kabekkodu SP Brand A : Perspectives on Translational Genomics and Public Health in India. *Public Health Genomics.* 2016;19(2):61–8. 10.1159/000442518 26683060

[9] Indian Genome Variation Consortium: The Indian Genome Variation database (IGVdb): a project overview. *Hum Genet.* 2005;118(1):1–11. 10.1007/s00439-005-0009-9 16133172

[10] Narang A Roy RD Chaurasia A : IGVBrowser--a genomic variation resource from diverse Indian populations. *Database (Oxford).* 2010;2010:baq022. 10.1093/database/baq022 20843867 PMC2942067

[11] Rajagopal D : India to launch its 1st human genome cataloguing project - The Economic Times. 2019. Reference Source

[12] IndiGen: Genomics for Public Health - About. Reference Source

[13] Department of Biotechnology: Human Genetics & Genome Analysis. Department of Biotechnology. Reference Source

[14] Indian Council of Medical Research: National Ethical Guidelines for Biomedical and Health Research Involving Human Participants.ICMR; New Delhi.2017. Reference Source 10.4103/picr.PICR_10_18 PMC6647898 31404208

[15] Department of Biotechnology and Ministry of Science & Technology: Biological Data Storage, Access and Sharing Policy Of India.Government of India. 2019. Reference Source

[16] Government of India: The Personal Data Protection Bill.Bill No. 373 of 2019. Lok Sabha. 2019. Reference Source

[17] Government of India: The DNA Technology (Use and Application) Regulation Bill.Bill No. 128 of 2019. Lok Sabha. 2019. Reference Source

[18] Vaz M Vaz MKS : Ethical challenges in biobanking: moving the agenda forward in India. *Indian J Med Ethics.* 2014;11(2):79–88. 10.20529/IJME.2014.022 24727618

[19] Vaz M Vaz M Srinivasan K : Listening to the voices of the general public in India on biomedical research--an exploratory study. *Indian J Med Ethics.* 2015;12(2):68–77. 10.20529/IJME.2015.024 25920970

[20] Vaz M Sridhar TS Pai SA : The ethics of research on stored biological samples: outcomes of a Workshop. *Indian J Med Ethics.* 2016;1(2):118–22. 10.20529/IJME.2016.032 27260824

[21] Vaz M Palmero AG Nyangulu W Diallo AA Ho CWL. : Diffusion of ethical governance policy on sharing of biological materials and related data for biomedical research [version 1; peer review: 1 approved, 1 approved with reservations]. *Wellcome Open Res.* 2019;4: 170. 10.12688/wellcomeopenres.15480.1

[22] Barbosa S Pare Toe L Thizy D : Engagement and social acceptance in genome editing for human benefit: Reflections on research and practice in a global context [version 2; peer review: 4 approved]. *Wellcome Open Res.* 2021;5:244. 10.12688/wellcomeopenres.16260.2 34095505 PMC8142603

[23] Government of India: The Citizenship (Amendment) Act.Act No. 47 of 2019. Ministry of Law and Justice; New Delhi, 2019.

[24] Jonnalagadda K : The Aadhaar ecosystem leaks too much data. mint. 2018. Reference Source

[25] Khaira R : Rs 500, 10 minutes, and you have access to billion Aadhaar details.Tribuneindia News Service. 2018. Reference Source

[26] Joshi D : The UIDAI Has No Authority to Verify Indian Citizenship.The Wire,2020. Reference Source

[27] The Wire Analysis: With Notices to “Illegal Immigrants”, UIDAI Raises Two Key Questions.The Wire,2020. Reference Source

[28] Goisauf M Durnová AP : From engaging publics to engaging knowledges: Enacting "appropriateness" in the Austrian biobank infrastructure. *Public Underst Sci.* 2019;28(3):275–89. 10.1177/0963662518806451 30324869

[29] Gale NK Heath G Cameron E : Using the framework method for the analysis of qualitative data in multi-disciplinary health research. *BMC Med Res Methodol.* 2013;13:117. 10.1186/1471-2288-13-117 24047204 PMC3848812

[30] Vaz M Vaz M Srinivasan K : The views of ethics committee members and medical researchers on the return of individual research results and incidental findings, ownership issues and benefit sharing in biobanking research in a South Indian city. *Dev World Bioeth.* 2018;18(4):321–30. 10.1111/dewb.12143 28513968

[31] Guest G Bunce A Johnson L : How Many Interviews Are Enough?: An Experiment with Data Saturation and Variability. *Field Methods.* 2006;18(1):59–82. 10.1177/1525822X05279903

[32] O'Doherty KC Burgess MM Edwards K : From consent to institutions: designing adaptive governance for genomic biobanks. *Soc Sci Med.* 2011;73(3):367–374. 10.1016/j.socscimed.2011.05.046 21726926

[33] Lemke AA Harris-Wai JN : Stakeholder engagement in policy development: challenges and opportunities for human genomics. *Genet Med.* 2015;17(12):949–57. 10.1038/gim.2015.8 25764215 PMC4567945

[34] Chennells RS : Equitable Access to Human Biological Resources in Developing Countries: Benefit Sharing Without Undue Inducement.Springer,2016. Reference Source

[35] Beaton A Hudson M Milne M : Engaging Māori in biobanking and genomic research: a model for biobanks to guide culturally informed governance, operational, and community engagement activities. *Genet Med.* 2017;19(3):345–51. 10.1038/gim.2016.111 27632687

[36] Moodley K Beyer C : Tygerberg Research Ubuntu-Inspired Community Engagement Model: Integrating Community Engagement into Genomic Biobanking. *Biopreserv Biobank.* 2019;17(6):613–24. 10.1089/bio.2018.0136 31603696 PMC6921246

[37] Schicktanz S Schweda M Wynne B : The ethics of 'public understanding of ethics'--why and how bioethics expertise should include public and patients' voices. *Med Health Care Philos.* 2012;15(2):129–39. 10.1007/s11019-011-9321-4 21448745 PMC3319876

[38] Gehlert S Mozersky J : Seeing Beyond the Margins: Challenges to Informed Inclusion of Vulnerable Populations in Research. *J Law Med Ethics.* 2018;46(1):30–43. 10.1177/1073110518766006 30093794 PMC6077979

[39] Mithani Z Cooper J Boyd JW : Race, Power, and COVID-19: A Call for Advocacy within Bioethics. *Am J Bioeth.* 2021;21(2):11–8. 10.1080/15265161.2020.1851810 33289442

[40] Egorova Y : Castes of genes? Representing human genetic diversity in India. *Genom Soc Policy.* 2010;6(3):32. 10.1186/1746-5354-6-3-32

[41] Laurie G : What Does It Mean to Take an Ethics+ Approach to Global Biobank Governance? *ABR.* 2017;9:285–300. 10.1007/s41649-017-0030-z

[42] Vaz M : Extended Data for Respecting values and perspectives in biobanking and genetic research governance: Outcomes of a qualitative study in Bengaluru, India. Harvard Dataverse, V1,2022. 10.7910/DVN/ZUCAU5 PMC10357076 37485294

