# Supplementary material for: Respecting values and perspectives in biobanking and genetic research governance: Outcomes of a qualitative study in Bengaluru, India
Source: Wellcome Open Res. 2023 Mar 6;7:78. Originally published 2022 Mar 7. [Version 2] doi: 10.12688/wellcomeopenres.17628.2 (PMC10357076; doi:10.12688/wellcomeopenres.17628.2)
Supplement: Peer review history

# Peer review: Reviewer response for version 2

**DOI:** 10.21956/wellcomeopenres.21121.r61635

**Published:** 2023-07-25

**Authors:** Rita T Lawlor

**Affiliations:** 1 University and Hospital Trust of Verona, Verona, Italy

The paper is interesting and focuses on perspectives of biobanking and genetic research governance. The approach focused on in-depth interviews and focus group discussions. The number of participants in either group is extremely small and based on individual opinions and as stated by the authors is not representative of the demographic. The discussions were tailored to the focus group participant based on the first two focus groups and then fine-tuned therefore introducing some potential bias. Furthermore, these responses were received afterview of BGR and biobanks, again potentially introducing bias as the introduction had the potential to influence the type of response. This should be addressed in the limitations of the study with more information on how these discussion topics were fine tuned.

 The manuscript concerns biobank and BGR but these figures are barely represented particularly in the makeup of the topics in the interviews, and it is not indicated if they were involved in the development of the overview of the BGR. It would also be important to understand the origin of the video presented for clarity and transparency of the information provided before the discussions.

 Responses reported in the manuscript from IDI are anecdotal and cherry picked and it would have been interesting to have some statistical idea of common responses. This is lacking in the manuscript and would help in the discussion which has a long list of issues not specifically highlighted in the results section of the manuscript. Some issues highlighted in the results are not carried through to the discussion such as the Issues of collaboration and trust within the academic world. This is an issue mentioned by one of the IDIs and is worthy of comment in the discussion as it pertains to the process of collection and use of samples and data for research from participant to potential health advances.

Is the work clearly and accurately presented and does it cite the current literature?

Partly

If applicable, is the statistical analysis and its interpretation appropriate?

Not applicable

Are all the source data underlying the results available to ensure full reproducibility?

Partly

Is the study design appropriate and is the work technically sound?

Partly

Are the conclusions drawn adequately supported by the results?

Partly

Are sufficient details of methods and analysis provided to allow replication by others?

Partly

Reviewer Expertise:

Cancer research, genomic characterization, biobanking

I confirm that I have read this submission and believe that I have an appropriate level of expertise to confirm that it is of an acceptable scientific standard, however I have significant reservations, as outlined above.

# Peer review: Reviewer response for version 2

**DOI:** 10.21956/wellcomeopenres.21121.r61634

**Published:** 2023-07-19

**Authors:** Signe Mezinska

**Affiliations:** 1 University of Latvia, Rainis Boulevard, Latvia

The article provides qualitative analysis of ethical issues in governance of biobanking and genetic research in Bengaluru, India. In general, the article is topical and well-written. The authors have considerably improved the clarity of the manuscript to compare with the first version; however, some shortcomings are still present and the article might benefit from further amendments:

- As it was noted by previous reviewers, it would be important to clarify the current legal framework governing biobanks in India and gaps in this framework in the Introduction section (e.g., is there a unified definition of biobanks, should biobanks be registered, how are biobanks governed, is research ethics committee review required by law for biobank-based human subject research?) Although the authors now provide a list of legally binding documents and some guidelines, this list is descriptive and does not provide sufficient insight into content, gaps and implementation of the current legal framework.
  Also, the information provided is not always clear, for example, the following sentence on page 3 might need improvements: “Documentation of broad consent may be, combined with a multi-layered, multiple option consent format, especially for secondary use of stored samples/ datasets, with additional clauses on financial or non financial benefit sharing.” More information on content, gaps and implementation of the current legal framework might help the reader better understand concerns expressed by the participants of focus groups and interviews. Do the authors have any recommendations for improving the legal framework based on this research study that might be included in the Conclusions part? Are any changes in the legal framework recommended to implement the People-centred governance proposed as an optimal governance framework by the authors?
- On page 12 the authors introduce the “Framework for People-Centred Governance of BGR”. This framework includes five principles (transparency, accountability, public good, fairness and reciprocity); however, the article does not explain how these principles were chosen and the authors do not provide any references for the choice of principles. It would be important to provide an explanation on why and how these particular principles were chosen (and possibly also why some other ethical principles were not included, e.g., protection of individuals, participation and inclusion (see WMA Declaration of Taipei), non-maleficence, beneficence justice, respect for autonomy).
- Throughout the article anonymisation of data and ethical issues surrounding this process are mentioned several times. However, it would be important to discuss whether anonymisation of data always is in the best interests of a biobank donor, because anonymisation does not allow the return of individual results and incidental findings. Do the authors have suggestions on how to balance the benefits of anonymisation and the right to receive information on one’s health? Who and how should define and protect the best interests of biobank donors?
- The authors have not made the anonymised transcripts of focus groups and interviews available in open access. At the same time in the Data Availability section they state that "The Methods section contains detailed information to allow replication of the study." I would suggest to change or delete this sentence because the information included in the Methods section is clearly not sufficient for replication of the study. Availability of transcripts is often mentioned as one of the prerequisites for replication of qualitative studies and it is also recommended for increasing transparency of qualitative research (for example, see Aguinis & Solarino ^1^).

Is the work clearly and accurately presented and does it cite the current literature?

Partly

If applicable, is the statistical analysis and its interpretation appropriate?

Not applicable

Are all the source data underlying the results available to ensure full reproducibility?

Partly

Is the study design appropriate and is the work technically sound?

Yes

Are the conclusions drawn adequately supported by the results?

Yes

Are sufficient details of methods and analysis provided to allow replication by others?

Yes

Reviewer Expertise:

Biomedical research ethics; research participant rights; ethics in biobanking; sociology of medicine.

I confirm that I have read this submission and believe that I have an appropriate level of expertise to confirm that it is of an acceptable scientific standard, however I have significant reservations, as outlined above.

## References

[1] : Transparency and replicability in qualitative research: The case of interviews with elite informants. *Strategic Management Journal* .2019; 10.1002/smj.3015 10.1002/smj.3015

# Peer review: Reviewer response for version 1

**DOI:** 10.21956/wellcomeopenres.19500.r50323

**Published:** 2022-06-15

**Authors:** Ana Palmero

**Affiliations:** 1 Ministry of Health, Buenos Aires, Argentina

This is a well-written and relevant paper as public engagement in biobanking and genetic research governance development in LMICs has received increased attention during the past decade and it is still a challenging topic.

 This paper would be strengthened by addressing the issues mentioned hereafter. 

**Background:**

- The regulatory landscape of BGR in India is mentioned. The content of the regulations related to BGR should be described in more detail, particularly ethical aspects such as the informed consent process (e.g., is broad consent process accepted?), privacy and confidentiality issues, ethics review of studies, biobanks governance requirements, etc., in order to understand the regulatory landscape and what needs to be strengthened in the current regulation. Mentions to international ethics guidelines related to Biobanks could be included (e.g., CIOMS Guidelines, WMA Declarations of Taipei) as well.
- The authors state that a literature review was conducted “…on worldwide experiences with BGR ethics and governance that revealed that constructs of ‘co-production’, ‘engagement of knowledges’, ‘rules of engagement’ and ‘stewardship’ were useful in relation to BGR in India”. The findings of the review should be discussed and referenced, and the concepts mentioned and how/why they would be useful to BGR in India should be defined.

**Methods: **

- Demographic characteristics of the participants could be described (e.g., gender, age, education level, occupation) to allow understanding of the sample representativity. 
- On which basis were the topics for discussion in FGDs and IDIs defined?

**Results:**

- Many of the key concepts used in this section related to ethics and governance of BGR should be discussed and defined in the Introduction (e.g., the type of informed consent, oversight, governance structure, risks, benefit-sharing)

Is the work clearly and accurately presented and does it cite the current literature?

Partly

If applicable, is the statistical analysis and its interpretation appropriate?

Not applicable

Are all the source data underlying the results available to ensure full reproducibility?

Partly

Is the study design appropriate and is the work technically sound?

Yes

Are the conclusions drawn adequately supported by the results?

Yes

Are sufficient details of methods and analysis provided to allow replication by others?

Yes

Reviewer Expertise:

Bioethics, research ethics, Health and research policy

I confirm that I have read this submission and believe that I have an appropriate level of expertise to confirm that it is of an acceptable scientific standard, however I have significant reservations, as outlined above.

# Response

**Published:** 2022-12-08

**Authors:** Manjulika Vaz

Dear Dr Palmero, Thank you so much for reviewing this article and for your detailed constructive feedback. I have addressed your suggestions and will be uploading the revised version shorlty. I hope to have strengthened the readibility and the usability of the paper so that others in the field can be enabled to look at people centred governance of stored samples and data and public engagement, especially in LMICs. Thank you once again. I look forward to your final review once the new version is uploaded. Kind regards, Manjulika Vaz Bengaluru, India

# Response

**Published:** 2023-02-13

**Authors:** Manjulika Vaz

Dear Dr Palmero,

 My co-authors and I have worked on the manuscript addressing your insightful comments and have submitted Version 2. Specific responses to each of your comments are presented below.

 Thank you for stating that this is a well-written and relevant paper as public engagement in biobanking and genetic research governance development in LMICs has received increased attention during the past decade and it is still a challenging topic. This paper would be strengthened by addressing the issues mentioned hereafter.

**Response** – Version 2 of the paper has included the modifications suggested towards its strengthening.

**Background:** The regulatory landscape of BGR in India is mentioned. The content of the regulations related to BGR should be described in more detail, particularly ethical aspects such as the informed consent process (e.g., is broad consent process accepted?), privacy and confidentiality issues, ethics review of studies, biobanks governance requirements, etc., in order to understand the regulatory landscape and what needs to be strengthened in the current regulation. Mentions to international ethics guidelines related to Biobanks could be included (e.g., CIOMS Guidelines, WMA Declarations of Taipei) as well.

**Response **– On page 4 of the Introduction details have been added from the Indian regulations and international regulations, as follows “ *While drug trials are governed by the Drugs and Clinical Trials rules under the 1940 Act , there is no specific law on BGR in India.* *The NEG 2017, compared to the 2006 NEG, has an entire chapter dedicated to Biological Materials, Biobanking and Datasets. This provides detailed descriptions of various types of consent processes (including blanket, broad, specific, dynamic, reconsent) and waivers. Documentation of broad consent may be combined with a multi-layered, multiple option consent format, especially for secondary use of stored samples/ datasets, with additional clauses on financial or non-financial benefit sharing. To safeguard confidentiality and privacy of donors of biological material and data, the practices of anonymization, coding and de-identification have also been discussed. In addition to ethical review and oversight by Research Ethics Committees (RECs), the NEG mandates that biobanks have a governance structure with a ‘technical authorisation committee’ to oversee the collection and disbursement of stored biological materials and data to researchers 14 . In spite of these explanations, much ambiguity remains owing to the complexities of different research designs that could be adopted to promote ethical use of and sharing of benefits from BGR 18– 20 . Stakeholder engagement could be a means that researchers and RECs could adopt to shape and implement research through the involvement of a wider group of interested stakeholders 21, 22 . In this connection, the Council for International Organizations of Medical Sciences (CIOMS)’s 2016 Guidelines 3, 7 and 8 highlight the importance of equitable distribution of benefits and burdens through community engagement and ethical collaborative arrangements. Where community engagement is applicable, there is an added responsibility on researchers involved in BGR to take into account participating communities’ values and preferences in research design and implementation 14 . Apart from consent and community engagement, data sharing is also encouraged as a means to maximise utility of stored genetic data 15 . Additionally, the World Medical Association’s Declaration at Taipei on Research on Health Databases, Big Data and Biobanks highlights that vigilance is required to prevent misuse and abuse where biological materials and genetic data are subject to multiple and indefinite uses. Under DBT’s policy, researchers are expected to follow the guidelines established by the ICMR in research involving human participants, which are aligned with principles of data sharing and protections of international conventions. However, the implementation of these principles and related considerations within a specific socio-cultural context of India requires careful study given that stakeholders’ expectations and understanding of consent, privacy and confidentiality, secondary research use and data sharing, among other ethical requirements vary greatly across the Indian subcontinent."* Additional references for all the new material has been also provided.

 The authors state that a literature review was conducted “…on worldwide experiences with BGR ethics and governance that revealed that constructs of ‘co-production’, ‘engagement of knowledges’, ‘rules of engagement’ and ‘stewardship’ were useful in relation to BGR in India”. The findings of the review should be discussed and referenced, and the concepts mentioned and how/why they would be useful to BGR in India should be defined.

**Response **– This has been expanded on page 5 with the following additions: “ *To enable the exploration of trustworthy governance approaches for BGR in India which are embedded in a system of public engagement, we initially conducted a literature review on worldwide experiences with BGR ethics and governance (1 ). The review revealed valuable modes of collaborative knowledge generation that are culturally situated and engaged. These include co-production (3), engagement of knowledges (31), rules of engagement (4) and relational practice of stewardship as seen in examples in Ghana5, Kenya6 , and among Māoris7. Based on these insights, the study reported in this paper was subsequently conducted to understand how incorporating people's values and beliefs can inform policy making decisions and strengthen BGR governance within India*.” with the following additional references: O’Doherty KC, Hawkins AK, Burgess MM: Involving citizens in the ethics of biobank research: Informing institutional policy through structured public deliberation. Soc Sci Med. 2012; 75(9): 1604–11; Godard B, Marshall J, Laberge C: Community Engagement in Genetic Research: Results of the First Public Consultation for the Quebec CARTaGENE Project. Community Genet. 2007; 10: 147–58; Dirks LG, Shaw JL, Hiratsuka VY, et al.: Perspectives on communication and engagement with regard to collecting biospecimens and family health histories for cancer research in a rural Alaska Native community. J Community Genet. 2019; 10(3): 435–446; Tindana P, Campbell M, Marshall P, et al.: Developing the science and methods of community engagement for genomic research and biobanking in Africa. Glob Health Epidemiol Genom. 2017; 2: e13. Moodley K, Singh S: “It’s all about trust”: reflections of researchers on the complexity and controversy surrounding biobanking in South Africa. BMC Med Ethics. 2016; 17(1): 57; Ngui EM, Warner TD, Roberts LW: Perceptions of African-American Health Professionals and Community Members on the Participation of Children and Pregnant Women in Genetic Research. Public Health Genomics. 2014; 17(1): 23–32. Munung NS, Mayosi BM, de Vries J: Equity in international health research collaborations in Africa: Perceptions and expectations of African researchers. Rosenbloom JL, editor. PLoS One. 2017; 12(10): e0186237. Staunton C, Tindana P, Hendricks M, et al.: Rules of engagement: perspectives on stakeholder engagement for genomic biobanking research in South Africa. BMC Med Ethics. 2018; 19(1): 13. Dennis-Antwi JA, Ohene-Frempong K, Anie KA, et al.: Relation Between Religious Perspectives and Views on Sickle Cell Disease Research and Associated Public Health Interventions in Ghana. J Genet Couns. 2018; 28(1): 102–118. Marsh VM, Kamuya DM, Mlamba AM, et al.: Experiences with community engagement and informed consent in a genetic cohort study of severe childhood diseases in Kenya. BMC Med Ethics. 2010; 11: 13 Beaton A, Hudson M, Milne M, et al.: Engaging Māori in biobanking and genomic research: a model for biobanks to guide culturally informed governance, operational, and community engagement activities. Genet Med. 2017; 19(3): 345–351.

**Methods:** Demographic characteristics of the participants could be described (e.g., gender, age, education level, occupation) to allow understanding of the sample representativity. 

**Response**: Gender and age have been added to the table (Table 1, see below) and this line on education has been added to the 2nd paragraph under Participants. “ *All participants held tertiary level qualification.*”

**Table 1. Profile of participants.** FGD No. Area of expertise No. of participants *(M/F/O) Age (median)* IDI No. Area of expertise/ involvement with BGR *(Gender, Age range in years)* 1. Public policy and public health experts 9 (7/2) 57 years 1. Breast cancer researcher (F, 40-45) 2. Activists and NGO representatives 7 (2/4/1) 45 years 2. Bioinformatics / Cancer genomics researcher (M, 30-35) 3. Media and communications personnel 8 (0/8) 34.5 years 3. Biobank manager / immunology researcher / clinician (M, 45-50) 4. Lawyers and data protection advocates 6 (5/1) 36.5 years 4. Rare disease patient (F, 30-35) 5. Post-graduate students and PhD scholars 6 (1/5) 32 years 5. Population geneticist (F, 30-35) 6. Patient interest organisation members 8 (4/4) 36.5 years 6. Genetic counsellor (F, 50-55) 7. Clinician / IEC member (F, 65-70) TOTAL participants 44 (19/24/1) 44.5 years 7 (M=2, F=5) 

 On which basis were the topics for discussion in FGDs and IDIs defined?

**Response**: The topics for discussion in the FGDs and IDIs were decided by the objectives of the study and the areas gleaned from the review of literature. To help make this clear, on page 7 the following line has been added: “ *The topics in the guide were collaboratively developed by the research team to address the study objectives and were informed by the literature review’ (1)"*

**Results:** Many of the key concepts used in this section related to ethics and governance of BGR should be discussed and defined in the Introduction (e.g., the type of informed consent, oversight, governance structure, risks, benefit-sharing)

**Response**: These have been added to the Introduction on page 4, as described under Bullet point 2 in the Background section above.

 Is the work clearly and accurately presented and does it cite the current literature? Partly

**Response**: With more details and citations added to the Introduction and the Discussion, we hope that this has been addressed. Thank you for your time and for your meaningful comments.

 Sincerely, Manjulika

# Peer review: Reviewer response for version 1

**DOI:** 10.21956/wellcomeopenres.19500.r50474

**Published:** 2022-06-08

**Authors:** Krishna Ravi Srinivas

**Affiliations:** 1 Research and Information System for Developing Countries (RIS), New Delhi, Delhi, India

1. The sample size is not significant.  As all stakeholders do not seem to have been represented the number and diversity of participants is a limitation.
2. The coverage of the literature is not adequate particularly when the article discusses so many issues ranging from benefit-sharing to informed consent. The legal dimension deserves more attention.
3. Whether the current guidelines and frameworks are adequate and how are they implemented should have elicited more discussion.
4. Similarly, best practices and guidelines that supplement the current framework could have been discussed.
5. "The data associated with this article is in the form of interview transcripts and group discussion transcripts with multiple personal anecdotes and experiences which cannot be anonymised sufficiently. To ensure confidentiality and privacy of all participants as stipulated in the ethical approval (by the Institutional Ethics Committee of St John’s Medical College & Hospital, Ref No. 208/2019), the data are not made publicly available. Nevertheless, the data will be available to external researchers upon a reasonable request to the first author via an email with a proposal of how the data will be used and a non-disclosure compliance statement."
  These could be publicly made available after due process of anonymization and removal of any marker/indication that could enable identification

Is the work clearly and accurately presented and does it cite the current literature?

Partly

If applicable, is the statistical analysis and its interpretation appropriate?

I cannot comment. A qualified statistician is required.

Are all the source data underlying the results available to ensure full reproducibility?

Partly

Is the study design appropriate and is the work technically sound?

Yes

Are the conclusions drawn adequately supported by the results?

Yes

Are sufficient details of methods and analysis provided to allow replication by others?

Yes

Reviewer Expertise:

ELSI aspects of bio/lifesciences, IP and access, privacy, cross-border data flows, Digital Sequence Information (Law & Policy)

I confirm that I have read this submission and believe that I have an appropriate level of expertise to confirm that it is of an acceptable scientific standard.
